# Collection of environmental DNA from stemflow for monitoring arboreal biodiversity: Preliminary validation using lichens

**DOI:** 10.1016/j.mex.2023.102448

**Published:** 2023-10-18

**Authors:** Ayumi Sakata, Tetsuya Sado, Shin-ichiro Oka, Masayuki Ushio, Masaki Miya

**Affiliations:** a Natural History Museum and Institute, Chiba, Japan; b Okinawa Churashima Institute, Okinawa, Japan; c Hong Kong University of Science and Technology, Clear Water Bay, Kowloon, Hong Kong SAR, China

**Keywords:** eDNA, Lichen, Rainwater, Metabarcoding, Forest canopy, Environmental DNA collection method from stemflow

## Abstract

The forest canopy harbors a diverse array of organisms. However, monitoring their biodiversity poses challenges due to limited accessibility and the vast taxonomic diversity. To address these challenges, we present a novel method for capturing arboreal biodiversity by harnessing stemflow as a source of DNA from organisms inhabiting trees. Our method involves encircling the tree trunk with gauze, directing the stemflow along the gauze into a funnel, and collecting it in a plastic bag. We employed dual collection systems to retrieve environmental DNA (eDNA) from the stemflow: the gauze trap, designed to capture macroscopic biological fragments, and the plastic bag trap, which collected the stemflow itself. The trapped fragments and stemflow were separately filtered, and eDNA was subsequently extracted from the filter membranes. To validate our method, we focused on foliose lichens, which are easily observable on tree surfaces. We performed eDNA metabarcoding and successfully detected a majority of the observed foliose lichen species, including those not identified through visual observation alone.

• We have developed a non-invasive and straightforward method for monitoring arboreal biodiversity by collecting eDNA from stemflow, which has been validated using lichens for its efficacy.
• This cost-effective approach minimizes disruptions to tree ecosystems and is expected to provide an efficient means of sampling and monitoring arboreal organisms.

## Graphical abstract

**Figure:**
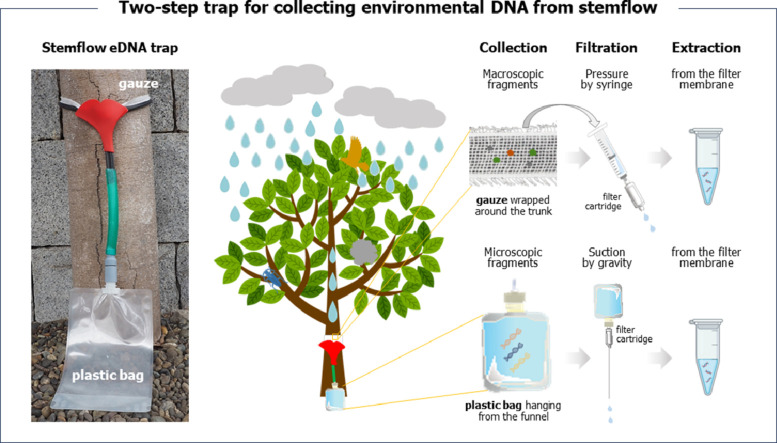

Specifications table

**Table:** 

Subject area:	Agricultural and Biological Sciences
More specific subject area:	Environmental DNA
Name of your method:	Environmental DNA collection method from stemflow
Name and reference of original method:	M. Miya, T. Minamoto, H. Yamanaka, S. Oka, K. Sato, S. Yamamoto, T. Sado, H. Doi, Use of a filter cartridge for filtration of water samples and extraction of environmental DNA, JoVE. (2016) No. 117. https://doi.org/10.3791/54741.
Resource availability:	A 3D-printed part file is available for download as Supplementary File

## Method details

Forest canopies are widely recognized for harboring a remarkable level of biodiversity [1]. However, monitoring the biodiversity in these habitats poses significant challenges due to limited accessibility and the vast taxonomic diversity of organisms that inhabit trees [2,3]. A variety of methods, such as steel towers, single-rope techniques, scaffolds, walkways, and construction cranes, have been developed to access and study canopy biodiversity [3], [4], [5], [6]. Despite these advancements, these methods often possess limitations in terms of flexibility, maneuverability, cost, and safety [3]. In fact, about 10 % of canopy researchers have reported difficulties in obtaining adequate samples for their studies [2]. This issue has led to a substantial knowledge gap regarding arboreal biodiversity in many locations, underscoring the urgent need for the development of more straightforward and efficient methods for comprehensive monitoring.

In this paper, we propose the use of stemflow, which refers to rainwater running down tree branches or trunks [7], as a potential source of environmental DNA (eDNA) shed by organisms inhabiting trees. eDNA has received considerable attention in recent years as a valuable tool for biodiversity monitoring [8]; however, its application to arboreal organisms has been limited [9], [10], [11]. To address this issue, we have developed a simple apparatus for collecting eDNA from these organisms present in the stemflow. Our method is non-invasive, obviating the need for organism collection, thereby minimizing environmental impact. The efficacy of our method was evaluated by filtering the collected stemflow through a filter membrane and extracting eDNA from the membrane. Additionally, we conducted eDNA metabarcoding, a technique that facilitates the simultaneous detection of multiple species. We specifically focused on foliose lichens, which are readily observable indicators of arboreal biodiversity on tree trunks and branches. This approach based on eDNA metabarcoding using stemflow is cost-effective, with an expenditure of only $80 per sample.

Our stemflow trap represents an improvement over the conventional method that utilizes gauze [12]. In our approach, gauze is wrapped around the tree trunk to guide the stemflow along its surface, subsequently directing it into a funnel that channels the flow into a collection bag. The installation of the stemflow collection system takes approximately 10 min. To recover eDNA from the stemflow, we implemented a dual-trap system. The first trap employs gauze to capture macroscopic fragments, while the second trap uses a separate plastic bag to collect microscopic fragments. This dual-trap approach is expected to enhance the efficiency of eDNA collection from lichens and other organisms.

**Fig. 1:**
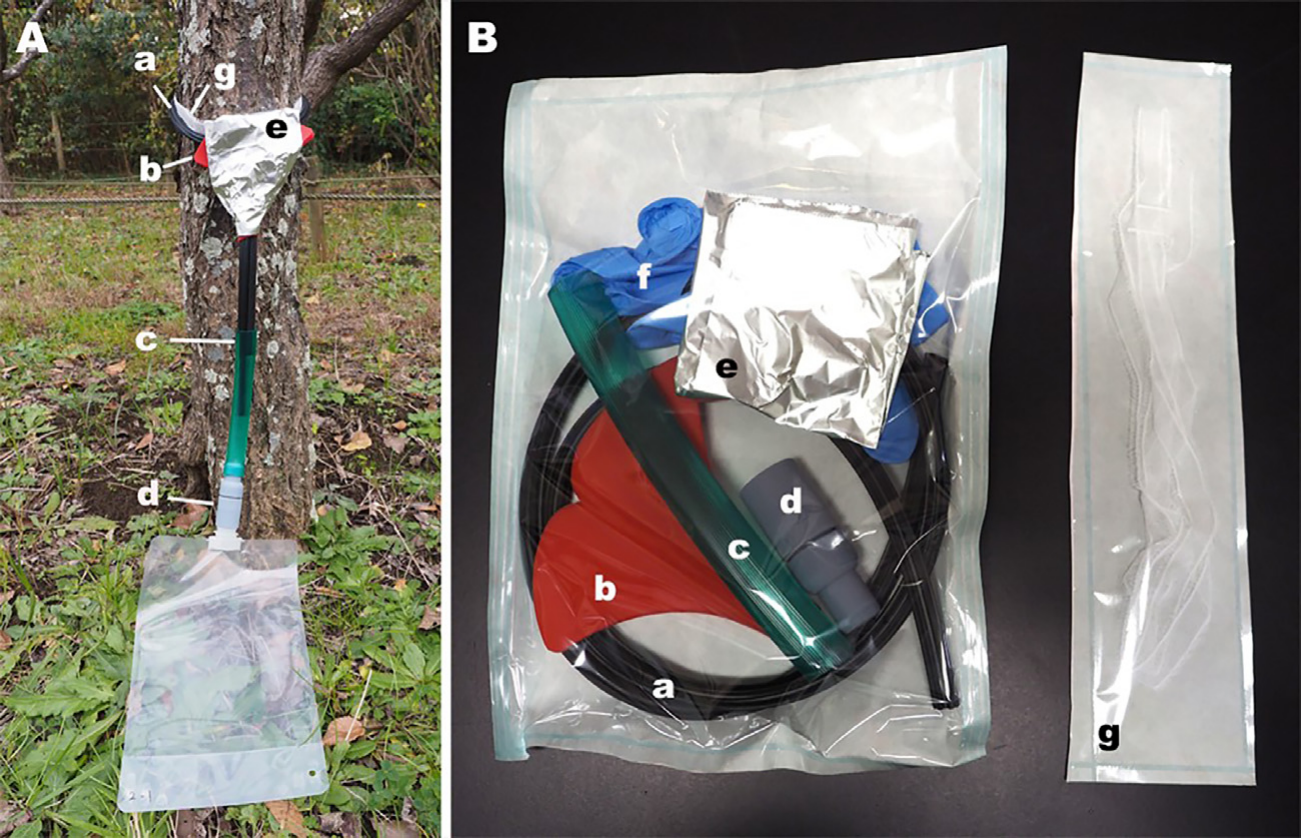
The stemflow recovery system (A) and its components (B). The components include the rubber rope (a), silicone funnel (b), hose (c), backflow prevention unit (d), aluminum foil (e), gloves (f), and gauze (g), all placed in sterilization packs to minimize contamination risk.

The trap consists of six components (Fig. 1): (1) A round rubber rope, with a circumference equal to the tree trunk plus 15–20 cm (IT-9149, Itodai Seiko), used for securing the gauze in place. (2) Gauze, approximately 1 m long (Insert Care Gauze No. 1, 63-1452-99, Hakujuji Co., Ltd), used for collecting macroscopic fragments. (3) A silicone funnel (Silicone Funnel Icho, 07,438, Yamazaki) that attaches to the rubber rope and guides the stemflow. (4) A hose, with an inner diameter of 1.5 cm and approximately 10 cm long, that connects the rubber rope to the backflow prevention unit. (5) A 1-L plastic bag (DP16-TN1000, Cowpack LTD) with an airtight screw cap, commonly used for gel or liquid foods, used for collecting the stemflow. (6) A backflow prevention unit, which ensures the stemflow does not reverse direction.

The workflow is as follows:

1. Securely tie a rubber rope around the tree trunk, approximately 50 cm above the ground level, to prevent the gauze from sliding down (Fig. 2A).

**Fig. 2:**
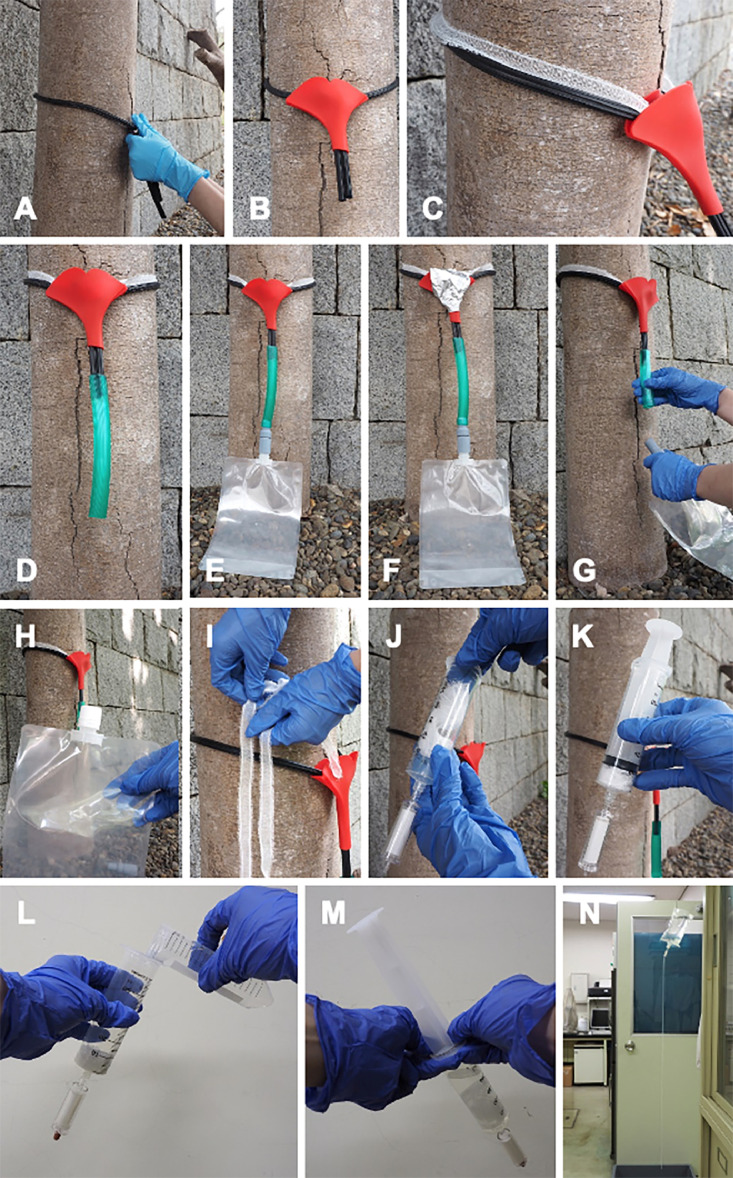
Setup protocol for the stemflow recovery system for eDNA sampling. (A) The rubber rope is securely tied around the tree trunk. (B) The ends of the rubber rope are threaded through a silicone funnel. (C) Gauze is laid along the tree trunk over the rubber rope, with its ends tucked into the silicone funnel. (D) Both ends of the rubber rope are inserted into the hose. (E) The hose is connected to the backflow prevention unit attached to a plastic bag. (F) The top of the funnel is covered with aluminum foil to minimize contamination. (G) Following rainfall, the plastic bag and the backflow prevention unit are detached from the hose. (H) The backflow prevention unit is removed from the plastic bag and capped (continue to step N). (I, J) The gauze is retrieved from the tree trunk and placed in a syringe fitted with a Sterivex filter cartridge. (K) The plunger is inserted into the syringe for transport indoors. (L) The plunger is removed, 50 mL of purified water is poured into the syringe, and the plunger is reinserted. (M) The syringe is shaken vigorously for 1 min, followed by pressure filtration. (N) Gravity filtration is performed using the stemflow collected in the plastic bag.
2. Thread both ends of the rubber rope through a silicone funnel to secure it in place (Fig. 2B).
3. Wrap the gauze around the tree trunk, ensuring that both ends of the gauze are inserted into the silicone funnel (Fig. 2C).
4. Insert both ends of the rubber rope into the hose, ensuring a secure connection (Fig. 2D).
5. Connect the hose to the backflow prevention unit, which is attached to the plastic bag (Fig. 2E).
6. Cover the top of the funnel with aluminum foil to prevent contamination (Fig. 2F).
7. After rainfall, detach the plastic bag, along with the backflow prevention unit, from the hose (Fig. 2G).
8. Remove the backflow prevention unit from the plastic bag, secure it with a cap, and refrigerate it prior to filtration (Fig. 2H; proceed to Step 15).
9. Retrieve the gauze from the tree trunk (Fig. 2I), and place it in a syringe attached to a Sterivex filter cartridge (Fig. 2J).
10. Insert the plunger into the syringe and transport it to a laboratory (Fig. 2K).
11. Remove the plunger, pour 50 mL of purified water into the syringe, and then reinsert the plunger (Fig. 2L).
12. Shake the syringe for 1 min and proceed with pressure filtration (Fig. 2M).
13. Repeat Steps 11 and 12, thereby achieving a total of 100 mL of filtration.
14. After filtration, add 2 mL of RNAlater (Thermo Fisher Scientific) to the cartridge through the inlet using a disposable pipette (E-243, Nihon Medical Science, Inc), and store it in a refrigerator until DNA extraction.
15. Perform gravity filtration with the collected stemflow in the plastic bag (Fig. 1N; for method details, see reference [13]).

**Fig. 3:**
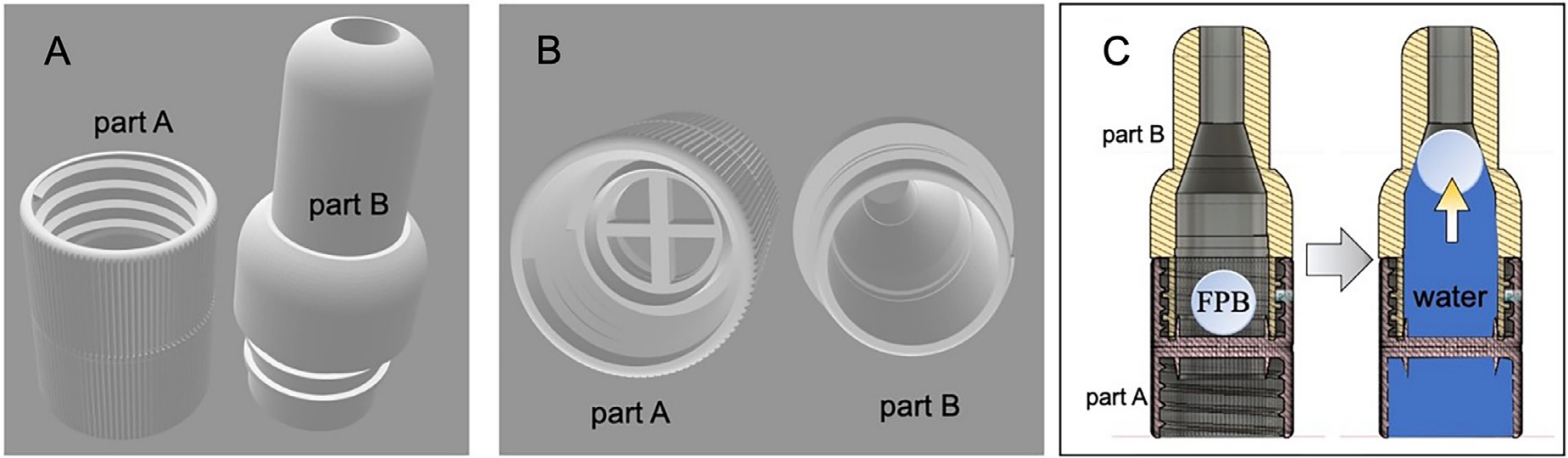
Top (A), bottom (B), and cross-section views (C) of the backflow prevention unit, designed using 3D CAD software, which can be connected to a sample bag and filter cartridge. FPB denotes a 1 cm diameter foam polystyrene ball. The 3D design file (in STL format) is available in the Supplementary materials.

The backflow prevention unit was designed using the 3D CAD software, "Fusion 360," to fit the plastic bag. It was then printed using an affordable and widely available stereolithography 3D printer, such as the ELEGOO Mars Pro 2 or ELEGOO Saturn. The unit consists of two parts (Fig. 3A, B): Part A, which connects to the plastic bag, and Part B, which attaches to the collection apparatus. Positioned between these two parts, there is a 1-cm diameter foam polystyrene ball (FPB) (Fig. 3C). As the stemflow fills the plastic bag, passing through this unit, the buoyancy of the polystyrene ball effectively prevents backflow by blocking the passage.

Below are several precautions to ensure the efficacy of this method:

• Prior to use, disinfect the rubber ropes, funnels, hoses, and backflow prevention units with 10 % commercial bleach (approximately 0.6 % sodium hypochlorite) to prevent contamination.
• To further avoid contamination, place all parts of the equipment in sterilization packs (Fig. 1B).
• Install the stemflow collection system before the onset of rainfall.
• To minimize the risk of contamination, change gloves between handling each unit of the stemflow collection apparatus.
• When installing the trap, ensure that the bark and gauze are in close contact.
• Add 1 mL of Benzalkonium chloride (Osban; 4987123116046, Nihon Pharmaceutical Co., Ltd.) to the plastic bag before collecting stemflow to prevent degradation of eDNA and fragments.
• Collect the gauze in a syringe fitted with a Sterivex filter cartridge to avoid contamination.

## Method validation

We selected three individual trees (Trees 1–3) from a *Prunus mume* grove in Aoba-no-Mori Park, Chiba City, for our study (Table 1). We visually identified the foliose lichens on these trees without the use of any access tools, observing the tree trunk and parts of the canopy up to an approximate height of 3 m from the ground. Each tree was observed for approximately one hour, and identification was primarily visual occasionally aided by a loupe, based on morphology. Two species, *Dirinaria applanata* and *Kashiwadia orientalis*, were the most widespread, nearly covering the entire surfaces of the tree trunks. Three other species —*Flavoparmelia caperata, Parmotrema austrosinese*, and *Parmotrema tinctorum*— were relatively common, found sporadically on all three trees. The remaining two species, *Parmotrema clavulifera* and *Parmelinopsis minarum*, were either rare or very rare, only occasionally observed on the tree trunks.

**Table 1:** Foliose lichens visually observed on three individual *Prunus mume* trees, categorized into four arbitrary dominance ranks across the three trees (dominant, common, rare, and very rare).

Species	Tree 1	Tree 2	Tree 3	Dominance
*Dirinaria applanata*	✓	✓	✓	Dominant
*Kashiwadia orientalis*	✓	✓	✓	Dominant
*Flavoparmelia caperata*	✓	✓	✓	Common
*Parmotrema austrosinese*	✓	✓	✓	Common
*Parmotrema tinctorum*	✓	✓	✓	Common
*Parmotrema clavulifera*	✓	✓	✓	Rare
*Parmelinopsis minarum*	✓	×	×	Very rare

After conducting visual observations, we collected a total of 18 stemflow samples, comprising nine from the gauze and nine from the plastic bags, over three separate rainy days from the three trees. We then filtered the stemflow using the methods described above (for detailed sampling data, see Table 2). Subsequently, we removed RNAlater from the filter cartridges, and homogenized the lichen fragments trapped on the filters [14], and performed DNA extraction. We extracted the eDNA from the filter membranes using the methods described by Miya et al. (2016) [15], with minor modifications. We used these 18 eDNA samples as templates for the subsequent metabarcoding analysis [8].

**Table 2:** Summary of sampling data. Note that a volume of 100 mL was filtered from each of the nine gauze samples. The rainfall duration and total rainfall were observed using a weather observation device (Shimazu Z-U14-50401), located 300 m away from the sampling site.

Tree No.	Rainfall times (hrs)	Total rainfall (mm)	Volume of stemflow (mL)	Volume of filtration (mL)	Date in 2022	Time
Tree 1	13	34.5	140	140	Oct.7–8	08:15–09:15
Tree 2			1180	390		
Tree 3			1340	340		
Tree 1	13	8.5	80	80	Oct.9–10	14:30–07:00
Tree 2			1490	220		
Tree 3			320	70		
Tree 1	17	31	110	110	Nov. 22–24	14:30–09:15
Tree 2			980	160		
Tree 3			860	160		

We utilized a two-step PCR approach to prepare paired-end libraries for metabarcoding analysis on the MiSeq platform [8]. We sequenced the library using a MiSeq Reagent Kit v2 with 150 bp x 2 PE. In the first-round PCR (1st PCR), we developed two sets of PCR primers to specifically designed to amplify fragments (approximately 170 bp) from the nuclear ribosomal internal transcribed spacer (ITS) regions of foliose lichens and not those of other fungi (Table 3). During the primer design process, we considered several technical tips to enhance primer annealing to the template without the use of degenerate bases [16] with the aid of Primer-BLAST [17]. We multiplexed these primers in a single 1st PCR reaction. In the second-round PCR (2nd PCR), we added unique combinations of indices and two adapter sequences to both ends of the amplified fragments for each sample. We then combined the 18 indexed samples into a single pool and subjected the pooled sample to sequencing on the MiSeq platform.

**Table 3:** PCR primer sequences used in this study.

Primer	Sequence (5´–3´)
ITS-PALichenF	ACACTCTTTCCCTACACGACGCTCTTCCGATCTNNNNNNCAACTCTTCACCCTGTGTGT
ITS-PALichenR	GTGACTGGAGTTCAGACGTGTGCTCTTCCGATCTNNNNNNATCGCATTTCGCTGCGTTCT
ITS-PHLichenF	ACACTCTTTCCCTACACGACGCTCTTCCGATCTNNNNNNTCTAACCGGCCTCAACTCTT
ITS-PHLichenR	GTGACTGGAGTTCAGACGTGTGCTCTTCCGATCTNNNNNNGATGCCAGAACCAAGAGATC

A total of 2,304,710 raw reads were generated from the MiSeq sequencing. These raw MiSeq reads underwent preprocessing to eliminate erroneous sequences and were analyzed using PMiFish [8]. The preprocessed dataset, comprising 2,073,013 reads, was subjected to taxonomic assignments using a sequence identity threshold of 98 % against the reference sequences. The reference sequences used for taxonomic assignments were compiled from the public sequence database, in addition to the original sequences (see Supplementary materials). After rarefaction, all minor molecular taxonomic units (MOTUs) with read counts of less than 0.01 % of the total reads (<12 reads) were excluded from the taxonomic table to ensure conservative estimates of MOTUs diversity. To refine the taxonomic assignments, generic-level phylogenies were reconstructed from MOTUs and reference sequences belonging to the respective genera, and the taxonomic table was revised following the method described in reference [18]. All raw DNA sequence data and associated information have been deposited in the DDBJ/EMBL/GenBank database and are available under accession number DRA 016873.

Of the seven species visually observed across the three trees, six species were detected via eDNA metabarcoding (Table 4). The lichen species were grouped into four categories, based on their frequency and coverage as determined by visual observation: dominant, common, rare, and very rare (Table 1). The two “dominant” species, *Dirinaria applanata* and *Kashiwadia orientalis* (Table 1), were detected in all nine gauze and nine plastic bag samples. Among the remaining species, three “common” species (*Flavoparmelia caperata, Parmotrema austrosinese*, and *Parmotrema tinctorum*) and one “rare” species (*Parmotrema clavuliferum*) were more frequently detected in the gauze samples (2–9 samples) compared to the plastic bag samples (0–5 samples). Notably, the “very rare” species (*Parmelinopsis minarum*) was not found in any of the 18 samples; however, two additional species (*Hyperphyscia adglutinata* and *Phaeophyscia* cf. *rubropulchra*) were exclusively detected through eDNA metabarcoding. It is worth noting that two non-targeted crustose lichens, *Amandinea punctata* and *Lecanora* sp., were only observed in the plastic bag samples. These findings suggest that our dual-trap approach was effective in comprehensively monitoring arboreal lichens. Consequently, the method exhibits potential for application in tropical forests with tall canopies and for monitoring the arboreal biodiversity of various organisms, including birds, insects, and other organisms.

**Table 4:** Summary of eDNA metabarcoding results for foliose lichens.

Species	Average identity	Total reads	Gauze sample	Plastic bag sample	Insertion length (bp)
*Dirinaria applanata*	98.3	926,132	9	9	174
*Kashiwadia orientalis*	97.7	1,032,891	9	9	145
*Flavoparmelia caperata*	96.6	258	3	0	149
*Parmotrema austrosinese*	100.0	95,229	9	5	145
*Parmotrema tinctorum*	100.0	13,940	4	1	145
*Parmotrema clavulifera*	99.7	170	2	0	147/172
*Hyperphyscia adglutinata*	97.4	3601	4	1	151
*Phaeophyscia* cf. *rubropulchra*	95.5	60	1	1	156

## Ethics statements

The field experiments conducted in Aoba-no-Mori Park were carried out with permission from the park administrator.

## CRediT authorship contribution statement

**Ayumi Sakata:** Conceptualization, Methodology, Investigation, Resources, Data curation, Writing – original draft, Funding acquisition. **Tetsuya Sado:** Validation, Data curation, Writing – review & editing. **Shin-ichiro Oka:** Methodology, Writing – review & editing. **Masayuki Ushio:** Conceptualization, Methodology, Writing – review & editing. **Masaki Miya:** Conceptualization, Methodology, Resources, Data curation, Writing – original draft, Supervision, Funding acquisition.

## Declaration of Competing Interest

The authors declare that they have no known competing financial interests or personal relationships that could have appeared to influence the work reported in this paper.

## Data Availability

We have deposited the DNA data in DDBJ and they are available freely.

## Appendix Supplementary materials

**File S1.** The 3D design file (STL) of the entire backflow prevention unit. Image, application 1

**File S2.** The 3D design file (STL) of the top of backflow prevention unit (part A). Image, application 2

**File S3.** The 3D design file (STL) of the bottom of backflow prevention unit (part B). Image, application 3

**Table S1.** Reference database used for taxonomic assignment. Image, application 4
